# Gastrointestinal presentation of COVID-19 in a pediatric heart transplant recipient

**DOI:** 10.1186/s43057-020-00034-z

**Published:** 2020-11-09

**Authors:** Abdelmonem Helal, Ahmed F. Elmahrouk, Ahmed A. Jamjoom, Jameel A. Al-Ata

**Affiliations:** 1 Pediatric Cardiology Section, Department of Pediatrics, King Faisal Specialist Hospital & Research Center, Jeddah, Kingdom of Saudi Arabia; 2 Department of Pediatrics and Pediatric Cardiology, Cairo University, Cairo, Egypt; 3 Division of Cardiac Surgery, Cardiovascular Department, King Faisal Specialist Hospital and Research Center, MBC J-16, P.O. Box: 40047, Jeddah, 21499 Saudi Arabia; 4 Cardiothoracic Surgery Department, Tanta University, Tanta, Egypt; 5 Section of Pediatric Cardiology, Department of Pediatrics, King Abdulaziz University, Jeddah, Saudi Arabia

**Keywords:** SARS-CoV-2, Pediatric heart transplant recipient, Gastrointestinal presentation

## Abstract

**Background:**

Severe acute respiratory syndrome coronavirus 2 (SARS-CoV-2)-associated coronavirus disease 2019 (COVID-19) most commonly causes a mild respiratory illness; however, there are wide ranges of presenting symptoms and disease severity. It has a mortality rate around 7%.

**Case presentation:**

We present a case of a 9-year-old female patient with hypoplastic left heart syndrome status post heart transplantation at age of 7 days. She presented to our emergency room complaining of intermittent fever, chills, fatigue, poor appetite, and diarrhea.

A throat swab nucleic acid test was positive for severe acute respiratory syndrome coronavirus 2 (SARS-CoV-2). Intravenous fluids therapy was used for correction of hydration status. To the best of our knowledge this is the first reported case of non-pulmonary presentation of coronavirus disease-2019 (COVID-19) in a pediatric heart transplant recipient, which was successfully managed conservatively.

**Conclusions:**

Gastrointestinal manifestations can be the only presenting symptom in pediatric heart transplant recipients with COVID-19. Conservative treatment could be used successfully. Immunomodulatory medications that are used in heart transplant recipients may have protective value in SARS-CoV-2 infection.

## Background

Severe acute respiratory syndrome coronavirus 2 (SARS-CoV-2)-associated coronavirus disease 2019 (COVID-19) most commonly causes a mild respiratory illness, however, there are wide ranges of presenting symptoms and disease severity [1]. It has a mortality rate around 7% [2].

Serious and fatal cases of COVID-19 have been recorded in children; however, most pediatric groups tend to have asymptomatic, mild, or moderate presentations. Most of them recover within 1 to 2 weeks of the onset of illness [3].

## Case presentation

A 9-year-old female patient was presented to our emergency room complaining of intermittent fever, chills, fatigue, poor appetite, and diarrhea. On examination, she was afebrile, (however, her family reported a tympanic temperature of 37.8 °C at home), oxygen saturation was 99% on room air, her respiratory rate was 20 breaths per minute with a heart rate of 90 beats/min, and her blood pressure measurement was 100/75 mmHg. The patient’s residential area is known to be a hotspot for SARS-CoV-2 infection. She had no history of similar manifestations in her family members.

### Past medical history

**Fig. 1:**
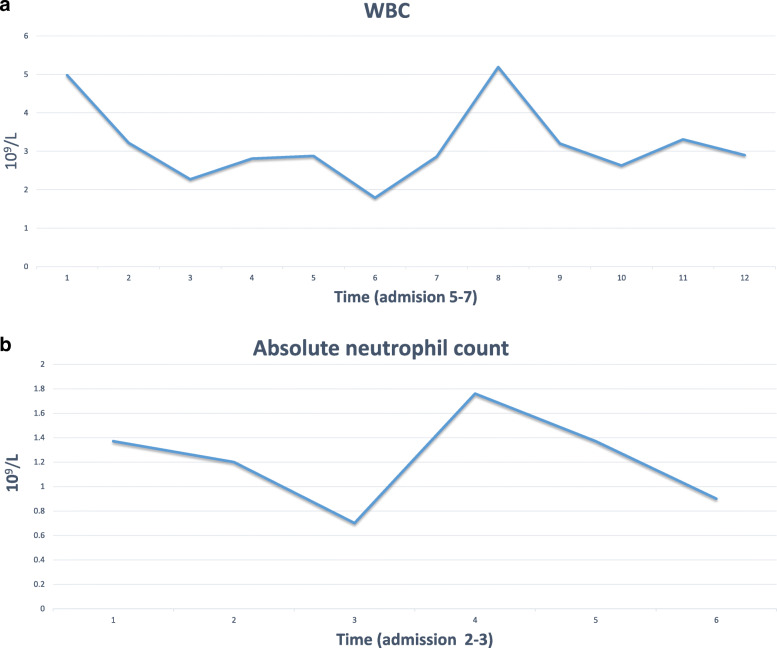
**a** A flow chart for leucocytic count (WBC) showing cyclic leucopenia prior COVID-19 infection, and a leucocytic count lower than her usual baseline during this admission, which started to recover by discharge. **b** A flow chart for absolute neutrophilic count showing neutropenia during COVID-19 infection, which started to recover by discharge

The child was born with hypoplastic left heart syndrome; she underwent a heart transplant operation at the age of 7 days. She had regular follow-up appointments in our pediatric cardiology clinic. Three years back, she was admitted to our facility with severe gastroenteritis attacks that required intensive care unit admission and intravenous inotropic support. After 10 days, she was discharged with stable hemodynamic conditions and normal cardiac functions. Last year, she was complaining about frequent attacks of eczema flare-up, which was treated successfully by a 5-day course of topical hydrocortisone 1% cream. Her current medications are aspirin 81 mg daily, immunosuppressive therapy in the form of tacrolimus 7 mg once daily, and mycophenolate mofetil 0.3 g three times a week. The last recorded blood concentration of tacrolimus 1 month before admission was 6 ng/ml, and cardiac allograft function was normal with an elevated baseline creatinine level of 70 umol/L and cyclic leucopenia with measured white blood cell count of 3.2 × 10^9^ cells/l and 5.3 × 10^9^ cells/l 4 and 2 months prior to this admission respectively (Fig. 1a). Absolute neutrophilic count was low on admission (0.3 × 10^9^ cells/l) and continue to rise to reach 2.5 × 10^9^ cells/l on follow-up (Fig. 1b).

### Differential diagnosis

The differential diagnosis included bacterial gastroenteritis, viral gastroenteritis, and coronavirus disease-2019 (COVID-19).

### Investigations

**Fig. 2:**
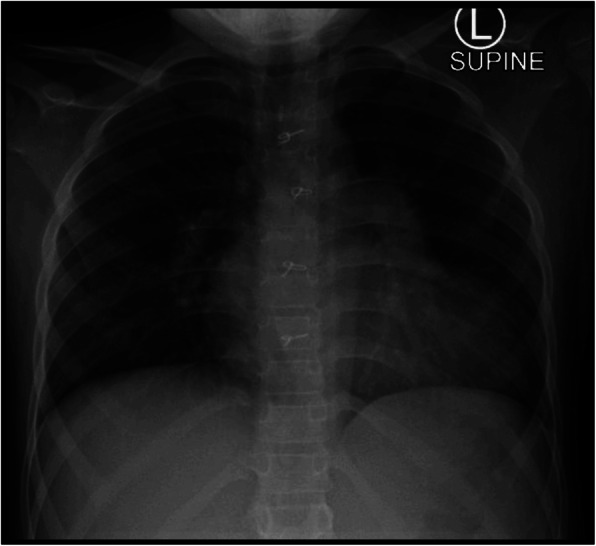
Chest X-ray showing that the cardiac silhouette is enlarged with the prominence of the left pulmonary artery. The lung fields and the costophrenic angles are clear

Laboratory tests showed a leucopenia (white blood cell count of 2.86 × 10^9^ cells/l) hemoglobin 9.2 g/dl, creatinine level 120 umol/l, renal Co2 12 mmol/l, procalcitonin level 8.4 ng/mL (normal range up to 2 ng/mL), D dimer 1.555 mg/l (normal range up to 0.555 mg/L), ferritin 229 μg/l, and ProBNP 680 pg/ml (normal range up to 450 pg/ml). Chest X-ray showed clear both lung fields and costophrenic angles. (Fig. 2).

**Fig. 3:**
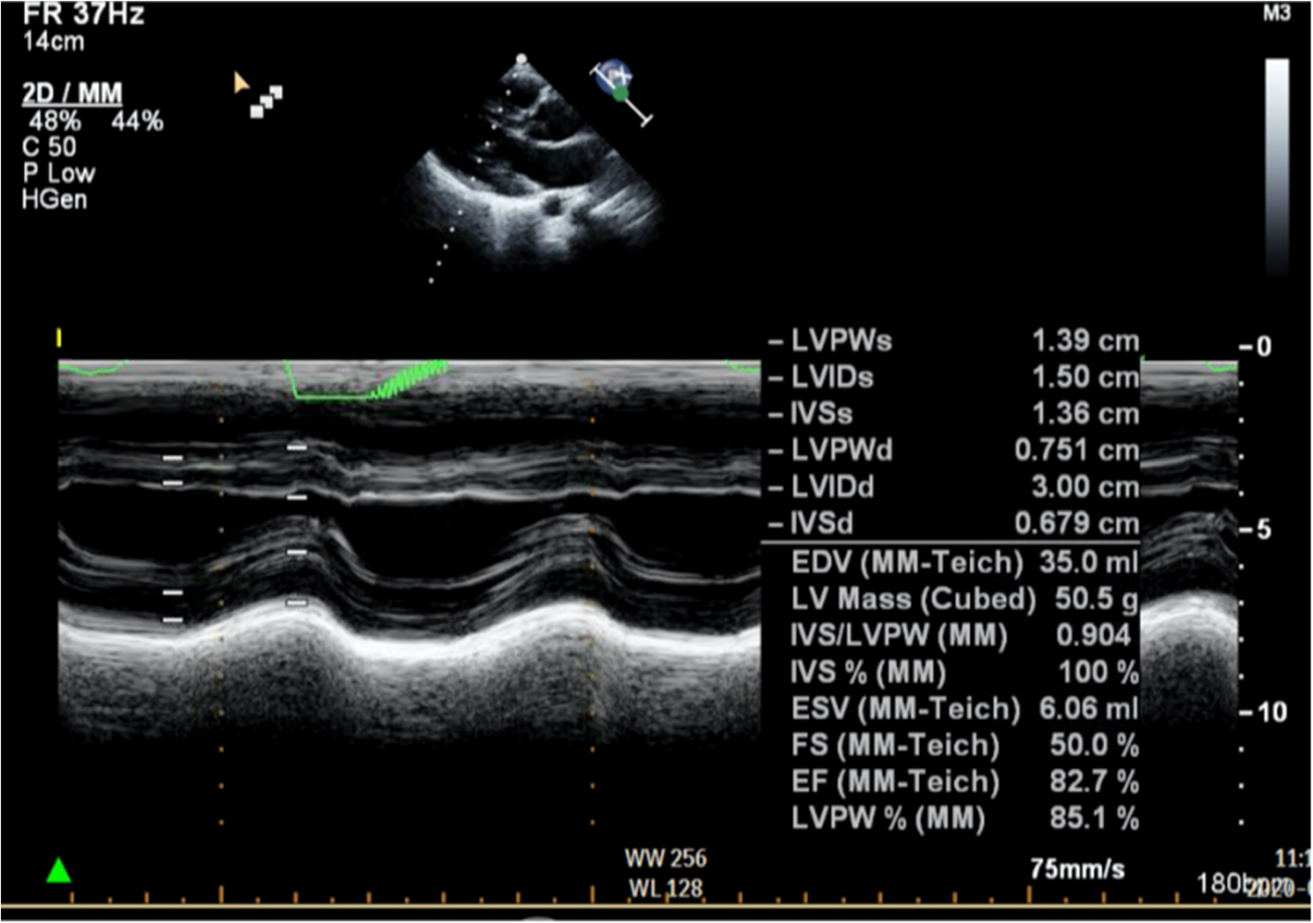
Echocardiography study showed normal systolic and diastolic function with no pericardial effusion

A throat swab nucleic acid test was positive for SARS-CoV-2. Vancomycin-resistant enterococci swab and methicillin-resistant Staphylococcus aureus swab screening were negative. Blood, urine, and stool cultures were negative for bacterial infection, and rapid antigen stool test was negative. Echocardiography study showed normal systolic and diastolic function with no pericardial effusion (Fig. 3).

### Management

**Fig. 4:**
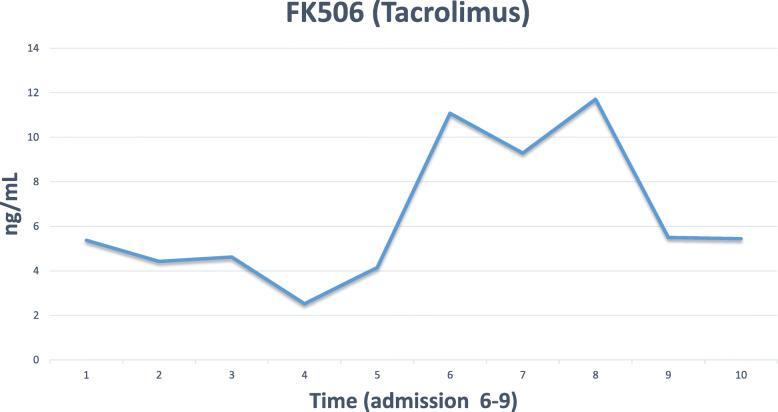
A flow chart of tacrolimus trough level showing an elevated level during the initial presentation of the disease, and the decrease of the level in response to dose adjustment

**Fig. 5:**
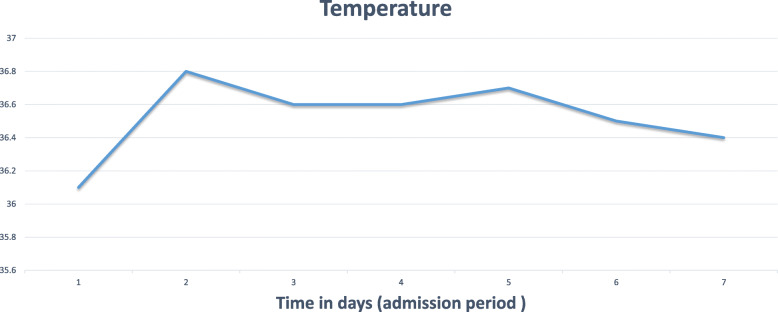
A temprature chart for recorded values throughout the admission period showing a highest recorded value of 37.5 °C

Immunosuppressive drugs were adjusted in the form of tacrolimus 5 mg as tacrolimus trough level concentration on admission was 11 ng/ml (Fig. 4) and continued on the same dose of mycophenolate mofetil and aspirin. Since the child condition was stable, a decision was made in a multi-disciplinary meeting between pediatric cardiology, cardiac surgery, pediatric infectious disease, and immunology to proceed with conservative management, and we did not start any antiviral medications for SARS-CoV-2 virus infection. Intravenous fluids therapy was used for the correction of hydration status. Body temperature was normal during the admission period (Fig. 5), gastrointestinal symptoms were gradually improving over three days, and she resumed her normal oral intake. On the 7th day of admission, laboratory tests showed creatinine level 70 umol/l, renal Co2 19 mmol/l. The patient was discharged after two consecutive negative reverse transcriptase−polymerase chain reaction throat swabs for SARS-CoV-2 on the 12th and 18th days from admission.

### Follow-up

The patient remained hospitalized for 15 days of isolation as per the ministry of health guidelines. She was scheduled for a follow-up appointment in the pediatric cardiology clinic.

## Discussion

Multiple reports suggested that pediatric COVID-19 patients experience not only less severe but different symptoms than adults. Their rate of hospitalization was 5.7% with less than 0.6% needed ICU admission, with mortality rate of 0.11% [4].

The severity of COVID-19 tends to be caused not only by viral invasion and proliferation but also by an extreme immune response characterized by cytokine storm, myocardial injury, and coagulopathy [5]. Questions were raised about whether heart transplant recipients may be at elevated risk of adverse outcomes with SARS-CoV-2 infection due to several specific comorbidities following cardiac transplantation, including hypertension and cardiac allograft vasculopathy [6]. In addition, while maintaining immunosuppression predisposes recipients to a higher risk of infection, immunosuppression has also been theorized as being protective against cytokine storms [7]. Moreover, an earlier study demonstrated that FK506 (Tacrolimus) inhibited the growth and replication of human coronaviruses SARS-CoV, at low, non-cytotoxic concentrations in cell culture [8]. Our patient was receiving tacrolimus at the time of infection; therefore, she may have a more efficient immune response than if she had received corticosteroid.

There have been conflicting reports about mortality among recipients of a heart transplant with COVID-19. While one report did not find any increase in mortality [1], another article showed 25% mortality in an adult cohort of 22 transplant recipients with SARS-CoV-2 infection [9].

In the case report by Russell MR et al. [10], the patient was admitted to receive intravenous immunoglobulin 1 week after surveillance laboratory studies showed anti-human leukocyte antigen antibody. Because of the history of cough, SARS-CoV-2 reverse transcriptase polymerase chain reaction test was performed and returned positive. They suggested that careful measurement of donor-specific antibodies be undertaken in heart transplant survivors of COVID-19 [10].

Our child had a good immunosuppressive balance, with no signs of rejection. Moreover, echocardiography study showed normal systolic and diastolic function with no pericardial effusion. For that, we did not feel the need to perform donor specific antibodies test.

Our patient had an acceptable course over the last 9 years. Although laboratory investigation showed increased levels of inflammatory markers, the clinical presentation was in a mild form with no significant signs of systemic viral manifestations or cytokine storm. With all previous factors, we decided not to start antiviral medication. Monitoring hemodynamic parameters and correction of hydration status were the main line of management. The patient improved slowly on this conservative treatment.

## Conclusions

Gastrointestinal manifestations can be the only presenting symptom in pediatric heart transplant recipients with COVID-19. Conservative treatment could be used successfully. Immunomodulatory medications that are used in heart transplant recipients may have protective value in SARS-CoV-2 infection. More studies are needed to evaluate the difference in clinical course and outcome between pediatric and adult heart transplant recipients with COVID-19.

## Data Availability

Data are available with the corresponding author upon request.

## Abbreviations

SARS-CoV-2: Severe acute respiratory syndrome coronavirus 2
COVID-19: Coronavirus disease-2019
